# Broad Antiviral Activity of Ginkgolic Acid against Chikungunya, Mayaro, Una, and Zika Viruses

**DOI:** 10.3390/v12040449

**Published:** 2020-04-15

**Authors:** Dalkiria Campos, Susana Navarro, Yessica Yadira Llamas-González, Madelaine Sugasti, José González-Santamaría

**Affiliations:** 1 Grupo de Biología Celular y Molecular de Arbovirus, Instituto Conmemorativo Gorgas de Estudios de la Salud, Panamá 0816-02593, Panama; dcampos@gorgas.gob.pa (D.C.); nsusana09@gmail.com (S.N.); qfb.y.llamas@gmail.com (Y.Y.L.-G.); madelaine313@gmail.com (M.S.); 2 Programa de Doctorado en Ciencias Biológicas, Universidad de la República, Montevideo 11200, Uruguay

**Keywords:** alphaviruses, chikungunya, Mayaro, Una, flavivirus, zika, ginkgolic acid, replication, inhibition

## Abstract

The alphaviruses Chikungunya (CHIKV), Mayaro (MAYV), Una (UNAV), and the flavivirus Zika (ZIKV) are emerging or re-emerging arboviruses which are responsible for frequent epidemic outbreaks. Despite the large impact of these arboviruses on health systems, there are no approved vaccines or treatments to fight these infections. As a consequence, there is an urgent need to discover new antiviral drugs. Natural products are a rich source of compounds with distinct biological activities, including antiviral properties. Thus, we aimed to explore the potential antiviral activity of Ginkgolic acid against the arboviruses CHIKV, MAYV, UNAV, and ZIKV. Viral progeny production in supernatants from cells treated or not treated with Ginkgolic acid was quantified by plaque-forming assay. Ginkgolic acid’s direct virucidal activity against these arboviruses was also determined. Additionally, viral protein expression was assessed using Western blot and immunofluorescence. Our results reveal that Ginkgolic acid promotes a dose-dependent decrease in viral titers in all tested viruses. Moreover, the compound demonstrated strong virucidal activity. Finally, we found that viral protein expression was affected by treatment with this drug. Collectively, these findings suggest that Ginkgolic acid could have broader antiviral activity.

## 1. Introduction

The *Alphavirus* (*Togaviridae* family) and *Flavivirus* (*Flaviviridae* family) genera are arthropod-borne viruses (arboviruses) with a widespread distribution in tropical and subtropical countries [1]. Both genera are enveloped single-stranded RNA viruses of positive polarity with genomes ranging from 10 to 12 kilobases in length [2,3]. Some members of these genera, including the Dengue (DENV), Chikungunya (CHIKV), and Zika (ZIKV) viruses, are efficiently transmitted by the same vector, mosquitoes of the *Aedes* genus [4,5]. Furthermore, in recent years, several members of these viral genera have emerged as important human pathogens that have caused severe epidemic outbreaks at a regional or global level [6].

Within the *Alphavirus* genus, 31 species of viruses have been identified and classified into 11 different complexes based on their antigenic characteristics [7,8]. According to this classification, CHIKV, Mayaro (MAYV) and Una (UNAV) have been grouped within the Selimki Forest antigenic complex [8]. Typically, these arthritogenic viral infections are associated with symptoms such as fever, headache, myalgia, retro-orbital pain, rash, joint pain and in some cases, limiting and long-lasting polyarthralgia [9,10]. Recently, severe clinical manifestations, including fatal outcomes, have been observed in patients infected with CHIKV, suggesting that some *Alphavirus* infections have the potential to produce serious disease [11,12].

On the other hand, the *Flavivirus* genus includes medically important pathogens such as DENV, Yellow fever virus (YFV), West Nile virus (WNV), Japanese encephalitis virus (JEV) and ZIKV [13]. Before its introduction in Brazil in 2015, ZIKV was thought to only cause mild infection [14]. However, following the outbreak in Brazil, ZIKV infection was correlated with serious neurological disorders, including microcephaly in newborns and the appearance of Guillain-Barré syndrome in adults [15]. Despite the large impact of these arbovirus infections on the health systems of affected countries and the frequent emergence of new viral pathogens, there are currently no approved treatments or vaccines to combat these infections. As a result, there is an urgent need to identify new broad-acting antiviral drugs.

With regard to identifying new potential drugs, natural products are a rich source of molecules with diverse biological activities [16]. Ginkgolic acid is a natural compound isolated from the seed coats or leaves of *Ginkgo biloba*, a plant that is widely used in traditional Chinese medicine [17]. Earlier reports have shown that Ginkgolic acid has antitumoral [18,19], antibacterial [20] and antiparasitic activity [21], as well as antiviral activity against human immunodeficiency virus (HIV) [22]. Moreover, this compound is able to inhibit SUMOylation, a post-translational protein modification that regulates key processes in the cell [23]. Nonetheless, Ginkgolic acid’s ability to disrupt arbovirus replication remains unexplored. The aim of this study was to evaluate the antiviral activity of Ginkgolic acid against the arboviruses CHIKV, MAYV, UNAV, and ZIKV.

## 2. Materials and Methods

### 2.1. Cell Line Cultures and Reagents

Vero (CCL-81), Vero-E6 (CRL-1586, both obtained from ATCC, Manassas, VA, USA) and HeLa cells (kindly provided by Dr. Carmen Rivas, CIMUS, Spain) were grown in Minimal Essential Medium (MEM) supplemented with 10% heat-inactivated fetal bovine serum (FBS), 2 mM of _L_-Glutamine and 1% penicillin-streptomycin antibiotic solution (Gibco, Waltham, MA, USA). Cell lines were cultured at 37 °C under a 5% CO_2_ atmosphere. Ginkgolic acid C15:1 [(Z)-6-(Pentadec-8-enyl)-2-hydroxybenzoic acid, Sigma-Aldrich, Saint Louis, MI, USA] was dissolved in Dimethyl sulfoxide (DMSO) at a 10 mM concentration and stored at -20 ºC until use. Working solutions of Ginkgolic acid were prepared in MEM at the indicated concentrations.

### 2.2. Virus Strains and Propagation

Mayaro (MAYV, AVR0565, San Martin, Peru) [24] and Una (UNAV, BT-1495-3, Bocas del Toro, Panama) [25] strains were obtained from the World Reference Center for Emerging Viruses and Arboviruses (WRCEVA) at University of Texas Medical Branch (UTMB), USA, and kindly provided by Dr. Scott Weaver. Chikungunya (CHIKV, Panama_256137_2014) [26] and Zika (ZIKV, 259249) strains were isolated from patient sera during Chikungunya and Zika outbreaks in Panama in 2014 and 2015, respectively. Virus strains were propagated, titrated, aliquoted and stored as previously described [27].

### 2.3. Cytotoxicity Analysis

Ginkgolic acid toxicity was assessed following the procedures of a previous study [27]. Briefly, 2.5 × 10^4^ Vero or HeLa cells grown in 96-well plates in MEM without phenol red were treated with DMSO or Ginkgolic acid at a concentration of 0, 1 or 10 µM for 24 h. Then, 5 mg/mL of 3-(4,5-Dimethyl-2-thiazolyl)-2,5-diphenyltetrazolium bromide (MTT, Sigma, Aldrich, Saint Louis, MI, USA) was added to the cells, and they were incubated for 4 h. Formazan crystals were dissolved in a solution of 4 mM HCl and 10% Triton X-100 in isopropanol, and absorbance was determined at 570 nm using a microplate reader spectrophotometer (BioTek, Winooski, VT, USA). Results are shown as the percentage of viable cells relative to untreated control cells.

### 2.4. Virus Plaque-Forming Assay

Viral titers in cell supernatants were quantified by plaque-forming assay as previously reported [27]. Briefly, 10-fold serial dilutions of infected cell supernatants were used to infect Vero-E6 (MAYV and UNAV) or Vero cells (CHIKV and ZIKV) grown in 6-well plates and incubated for 1 h at 37 °C. After virus absorption, the viruses were removed and the cells were overlaid with a solution of agar (1%) in MEM supplemented with 2% FBS and incubated for 3 days at 37 °C. Then, the agar was removed and the cells were fixed with 4% formaldehyde solution in PBS and stained with 2% crystal violet prepared in 30% methanol. Finally, the number of plaques was counted, and the viral titers were reported as plaque-forming units per milliliter (PFU/mL).

### 2.5. Viral Infection Assays

Viral infection experiments were performed in HeLa or Vero cells seeded in 12- or 24-well plates pre-treated with DMSO or Ginkgolic acid. We used a multiplicity of infection (MOI) of 1 or 10 for each virus tested, and the cells were incubated with DMSO or Ginkgolic acid at the indicated concentrations and times. Cell supernatants were harvested to quantify the viral titers using a plaque-forming assay. For the virucidal activity experiments, approximately 10^5^ or 10^6^ PFU of CHIKV, MAYV, UNAV or ZIKV were incubated in serum-free MEM with 10 µM of Ginkgolic acid for 1h at 37 °C. Then, the virus particles were quantified as stated above. Additionally, protein extracts from infected cells under the different experimental conditions were obtained for protein analysis.

### 2.6. Immunofluorescence Assay

Vero or HeLa cells cultivated on glass coverslips were infected with CHIKV, MAYV or UNAV at an MOI of 1. Following 24 h of infection, cells were fixed with 2% paraformaldehyde in PBS buffer for 20 min and permeabilized with 0.25% Triton-X100. Then, the cells were blocked in 2% bovine serum albumin solution in PBS for 20 min and stained overnight at 4 ºC with anti-CHIKV, anti-MAYV or anti-UNAV mouse ascitic fluid (kindly provided by Dr. Scott Weaver, WRCEVA-UTMB, USA). Afterward, cells were incubated with a Goat anti-mouse secondary antibody (Alexa Flour 488, Invitrogen, Carlsbad, CA, USA) for 1 h in the dark. Finally, coverslips were mounted on slides with Prolong Diamond Antifade Mountant with Dapi (Invitrogen, Carlsbad, CA, USA), and the images were captured with an FV1000 Flowview Confocal microscope (Olympus, Lombard, IL, USA). The pictures were analyzed with ImageJ software. The number of virus-positive cells were estimated in at least 10 fields and denoted as the percentage of positive cells.

### 2.7. Protein Analysis

Viral protein expression was assessed by Western blot as previously described [27]. Briefly, Vero or HeLa cells were infected with CHIKV, MAYV or UNAV and after 1 h of virus absorption, 10 µM Ginkgolic acid or 0.1% DMSO, both dissolved in fresh MEM, were added to the cells. Then, at different times post-infection, protein extracts were obtained in Laemmli buffer with 10% Dithiothreitol (Bio-Rad, Hercules, CA, USA). Proteins were fractionated in SDS-PAGE, transferred to nitrocellulose membranes and blocked in a 5% non-fat milk solution in T-TBS buffer for 30 min. Membranes were incubated overnight at 4 °C with the following primary antibodies: rabbit polyclonal anti-E1, rabbit polyclonal anti-nsP1 (both of which were previously validated against alphaviruses in our laboratory [27]), and mouse monoclonal anti-β-actin (8H10D10, Cat. # 3700, Cell Signaling Technology, Danvers, MA, USA). Next, the membranes were washed 3 times in T-TBS buffer and incubated with HRP-conjugated goat anti-rabbit (Cat. # 926-80011) or goat anti-mouse (Cat. # 926-80010) secondary antibodies (LI-COR, Lincoln, NE, USA) at room temperature for 1 h. Finally, the membranes were incubated with SignalFire^TM^ ECL Reagent (Cell Signaling Technology, Danvers, MA, USA) for 5 min and the quimioluminescent signal was detected with a C-Digit scanner (LI-COR, Lincoln, NE, USA).

### 2.8. Statistical Analysis

Data were analyzed with the Mann & Whitney test and One- or Two-way ANOVA test using GraphPad Prism Software version 8.4.0 for Mac. A p value < 0.05 was considered as statistically significant. All experiments were performed at least 3 times with 3 replicates. For each experiment, mean and standard deviation are shown.

## 3. Results

### 3.1. Ginkgolic Acid Reduces Alphavirus Progeny Yield

**Figure 1:**
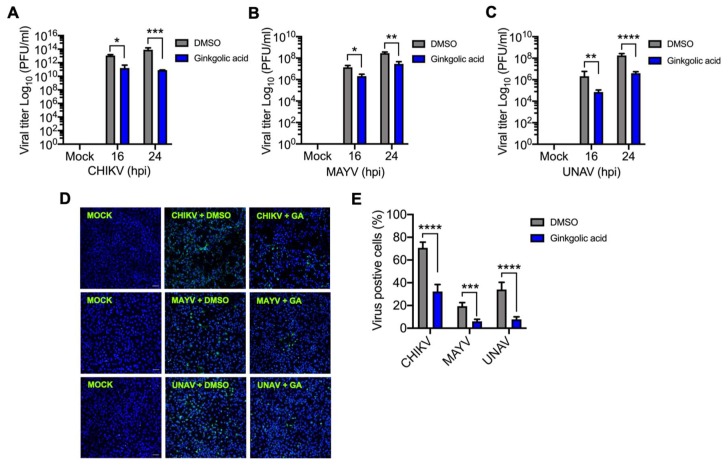
Ginkgolic acid impairs *Alphavirus* progeny production. Vero (**A**) and HeLa (**B**,**C**) cells were pre-treated with Ginkgolic acid at 10 µM or 0.1% DMSO as a control for 1 h. Then, the medium with the treatment was removed and the cells were infected with CHIKV, MAYV or UNAV at an MOI of 10. After 1 h of virus absorption, fresh medium was added with Ginkgolic acid or DMSO and the cells were incubated for 16 or 24 h post-infection (hpi). Viral progeny yield in cell supernatants collected at indicated times were quantified by plaque-forming assay. Viral titers were represented as plaque-forming units per milliliter (PFU/mL). Statistically significant differences were assessed with the Two-way ANOVA test: * *p* < 0.05; ** *p* < 0.01; *** *p* < 0.001 and **** *p* < 0.0001. (**D,E**) Vero or Hela cells grown on glass cover slips were pre-treated with Ginkgolic acid or DMSO and then infected with the *Alphavirus* at an MOI of 1. After 24 h of incubation with the treatment, cells were fixed and stained with anti-CHIKV, anti-MAYV or anti-UNAV mouse primary antibodies, followed by an Alexa-Flour 488 mouse secondary antibody. Cell nuclei were stained with Dapi (in blue). Images were obtained with a confocal microscope and analyzed with ImageJ software. The percentage of viral antigen-positive cells was evaluated in at least 10 fields for each experimental condition. These data were analyzed with the Two-way ANOVA test. Statistically significant differences are shown: *** *p* < 0.001 and **** *p* < 0.0001. Scale bar, 50 µm.

To examine the effect of Ginkgolic acid on alphavirus replication, we performed a kinetic infection in Vero or HeLa cells. The cells were pre-treated with Ginkgolic acid or DMSO for 1 h; then we removed the medium with the treatments, and the cells were infected with CHIKV, MAYV or UNAV. After 1 h of virus absorption, we added Ginkgolic acid or DMSO in fresh medium and viral progeny production in the cell supernatants was quantified by plaque-forming assay at the indicated times post-infection. As shown in Figure 1A–C, we observed a time-dependent increase in viral titers in DMSO-treated cells. On the other hand, in Ginkgolic acid-treated cells we found a significant reduction in viral yields for all tested viruses (Figure 1A–C). In order to confirm these results, we evaluated the presence of viral antigens in infected cells treated or not treated with Ginkgolic acid using immunofluorescence. These assays revealed that Ginkgolic acid treatment promotes a considerable decrease in the percentage of cells that demonstrate positive staining for the viral antigens (Figure 1D–E). To discard the possibility that the observed effect of Ginkgolic acid on viral replication was due to drug cytotoxicity, we assessed cell viability in Vero or HeLa cells incubated with different concentrations of the compound using the MTT method. In these experiments, we did not see a significant decline in cell viability after 24 h of incubation with Ginkgolic acid (Figure S1). Taken together, these results indicate that Ginkgolic acid affects the alphaviruses’ progeny yields.

### 3.2. Alphavirus Titer Decrease Is Ginkgolic Acid Concentration-Dependent

**Figure 2:**
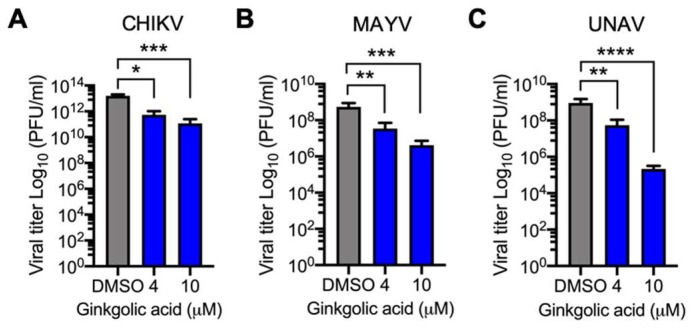
Ginkgolic acid’s effect on viral yield is concentration-dependent. Vero (**A**) and HeLa (**B**,**C**) cells were pre-treated with increasing concentrations of Ginkgolic acid and infected with CHIKV, MAYV or UNAV as previously described. After 24 h of incubation with the treatment or DMSO, production of infectious viral particles was quantified as previously described. Viral titers were represented as plaque-forming units per milliliter (PFU/mL). Experimental data were analyzed with the One-way ANOVA Test. Statistically significant differences are shown: * *p* < 0.05; ** *p* < 0.01; *** *p* < 0.001; **** *p* < 0.0001.

To evaluate whether *Alphavirus* progeny reduction is affected by drug concentration, we pre-treated Vero or HeLa cells with different concentrations of Ginkgolic acid before being infected with CHIKV, MAYV or UNAV, and the viral progeny production was measured as above. The viral progeny yields in DMSO-treated cells reached between 10^8^ and 10^12^ PFU/mL, depending on the *Alphavirus* being tested (Figure 2). However, in Ginkgolic acid-treated cells, we found a significant dose-dependent decrease in viral titers (Figure 2).

### 3.3. Virucidal Activity of Ginkgolic Acid against Alphavirus

**Figure 3:**
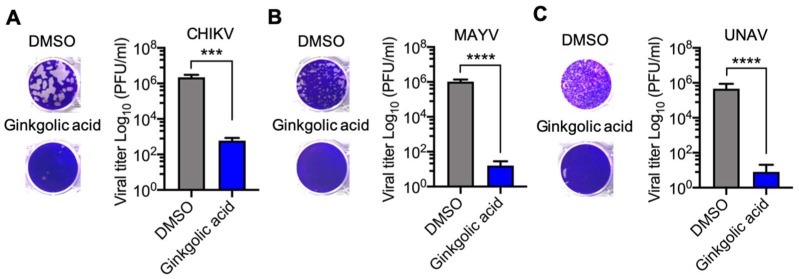
Ginkgolic acid alters *Alphavirus* particles’ infectivity. Approximately, 10^6^ PFU of CHIKV (**A**), MAYV (**B**) or UNAV (**C**) were incubated in serum-free medium with Ginkgolic acid at 10 µM or 0.1% DMSO as a control for 1 h at 37 ºC. Then, the alphaviruses were directly titrated in Vero or Vero-E6 cells and the remaining virus was quantified using a plaque-forming assay. Viral titers were represented as plaque-forming units per milliliter (PFU/mL). Data were analyzed using the Mann & Whitney test. Statistically significant differences are shown: *** *p* < 0.001; **** *p* < 0.0001.

In order to analyze if Ginkgolic acid has a direct effect on the infectivity of *Alphavirus* particles, we incubated 10^6^ PFU of CHIKV, MAYV or UNAV in serum-free medium with the Ginkgolic acid or DMSO at 37 °C for 1 h. Subsequently, the remaining virus in each experimental condition was quantified as previously indicated. As shown in Figure 3, Ginkgolic acid had a strong effect on viral infectivity; we found significant decreases of 4 to 5 logs in viral titers. These results suggest that Ginkgolic acid impairs the infectivity of *Alphavirus* particles.

### 3.4. Ginkgolic Acid Disrupts an Early Stage in the Alphavirus Replication Cycle

**Figure 4:**
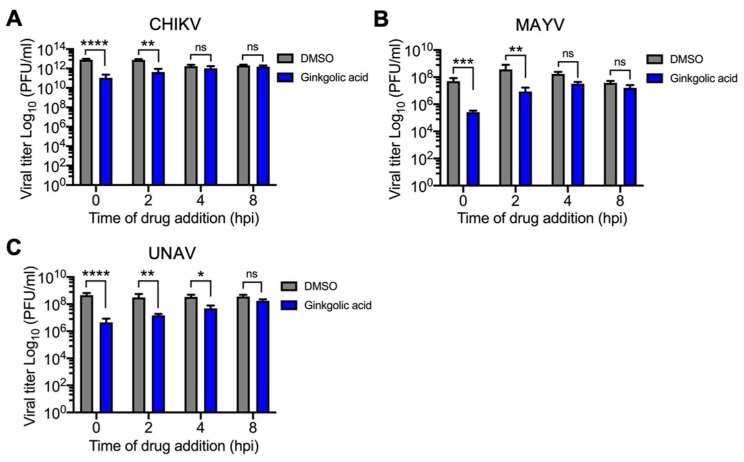
Ginkgolic acid prevents *Alphavirus* replication at an early stage of the viral cycle. Vero (**A**) or HeLa (**B**,**C**) cells were infected with CHIKV, MAYV, or UNAV and after 1 h of virus absorption, Ginkgolic acid or DMSO were applied to the cells at the indicated times. Then, the viral progeny production was measured in the cell supernatants after 24 h of incubation with the Ginkgolic acid or DMSO as previously indicated. The viral titers were expressed as plaque-forming units per milliliter (PFU/mL). Data were analyzed with the Two-way ANOVA test. Statistically significant differences are shown: * *p* < 0.05; ** *p* < 0.01; *** *p* < 0.001; **** *p* < 0.0001; ns: non-significant.

Given that we observed an antiviral effect when we pre-treated the cells or directly incubated the viruses with Ginkgolic acid, we decided to determine if this molecule is able to block *Alphavirus* replication after virus absorption. To investigate this, we performed time addition experiments in which we added Ginkgolic acid or DMSO at indicated times after 1 h of virus absorption (time 0 hpi); then we collected the cell supernatants at 24 hpi to measure viral progeny yields. These assays revealed that Ginkgolic acid only promoted a decline in *Alphavirus* progeny yields when the drug was applied in the first 2 hpi (Figure 4A–C). In the case of UNAV, we still noted a modest inhibitory effect at 4 hpi (Figure 4C). Altogether, these findings suggest that Ginkgolic acid inhibits *Alphavirus* replication at an early stage of the viral cycle.

### 3.5. Ginkgolic acid Suppresses the Expression of Alphavirus Proteins

**Figure 5:**
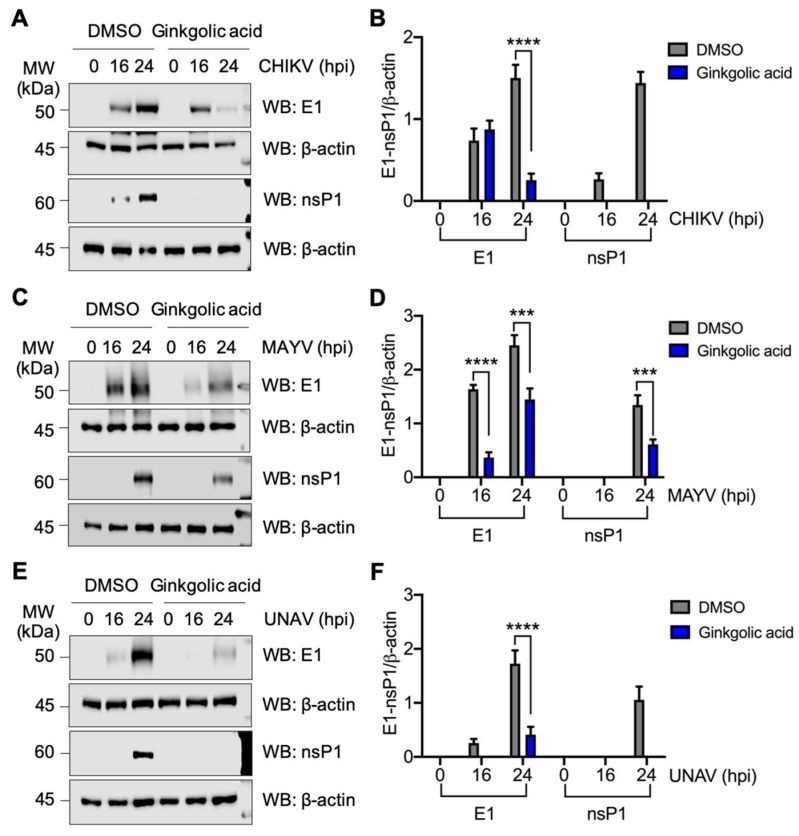
Ginkgolic acid affects alphavirus protein expression. Vero or HeLa cells were infected with CHIKV (**A**), MAYV (**C**), or UNAV (**E**) at an MOI of 10. After 1 h of virus absorption, Ginkgolic acid or DMSO were applied to the cells. At different times post-infection, the levels of E1 and nsP1 proteins were assessed by Western blot (WB). β-actin protein was used as a loading control. MW: Molecular weight. kDa: kilodaltons. **B**,**D**,**F**. Intensity bands for both E1 and nsP1 proteins were quantified using ImageJ software and normalized with β-actin protein. Data were analyzed with the Two-way ANOVA test. Statistically significant differences are shown: *** *p* < 0.001; **** *p* < 0.0001.

The previous results suggest that Ginkgolic acid could also have a negative effect on a post-entry step in the *Alphavirus* cycle. With respect to this, we wanted to explore other possible mechanisms by which Ginkgolic acid inhibits *Alphavirus* replication. Thus, we assessed the expression of viral proteins structural E1 and non-structural nsP1 in cell lysates obtained from cells infected with CHIKV, MAYV, or UNAV and treated with Ginkgolic acid or DMSO at different times. As shown in Figure 5A,C,E, we detected strong expression of both E1 and nsP1 proteins in DMSO-treated cells at 24 hpi for all evaluated alphaviruses. Conversely, Ginkgolic acid treatment resulted in a notable suppression of viral protein expression (Figure 5A,C,E). Furthermore, a semi-quantitative densitometric analysis for both viral proteins confirmed these findings (Figure 5B,D,F). Collectively, these results indicate that Ginkgolic acid affects the expression of *Alphavirus* proteins.

### 3.6. Ginkgolic Acid also Blocks Zika Virus Replication

**Figure 6:**
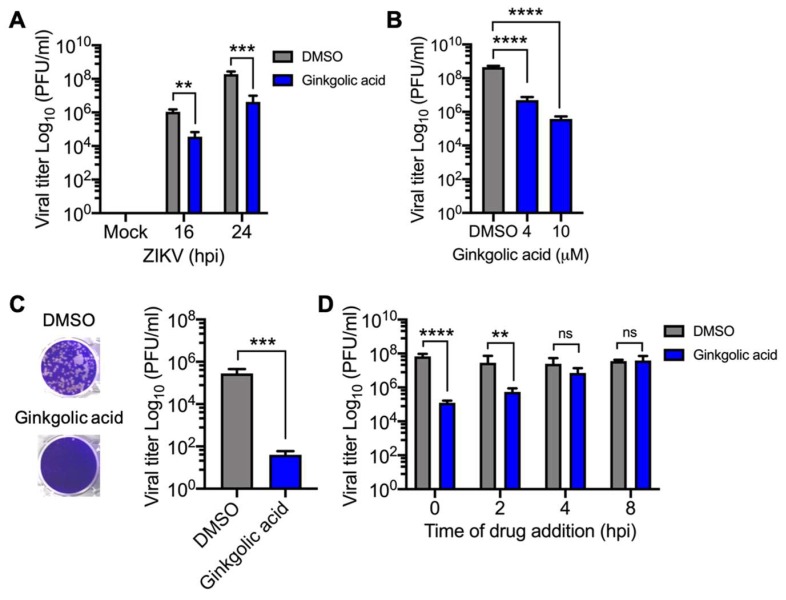
Ginkgolic acid also inhibits Zika virus (ZIKV) replication. (**A**) Vero cells were pre-treated with 10 µM Ginkgolic acid or 0.1% DMSO. After 1 h of incubation, the treatment medium was removed and the cells were infected with ZIKV at a multiplicity of infection (MOI) of 10. Then, fresh medium with Ginkgolic acid or DMSO was added to the cells and they were incubated for 16 or 24 hpi. Viral progeny production in the cell supernatants was measured as previously mentioned. (**B**) Vero cells were pre-treated with increasing concentrations of Ginkgolic acid and then infected with ZIKV as above. Viral titers in cell supernatants were quantified as previously described. (**C**) Nearly 10^5^ PFU of ZIKV were incubated in serum-free medium with Ginkgolic acid or DMSO as described previously. The remaining virus in both experimental conditions was titrated using plaque-forming assay. (**D**) Vero cells were infected with ZIKV; after 1 h of virus absorption, Ginkgolic acid was added to the cells at the indicated times. Viral production was evaluated as previously described. Viral titers were expressed as plaque-forming units per milliliter (PFU/mL). Data were analyzed with the One- or Two-way ANOVA test and Mann & Whitney test. Statistically significant differences are shown: ** *p* < 0.01; *** *p* < 0.001; **** *p* < 0.0001; ns: non-significant. **.**

Since our results suggested that Ginkgolic acid is able to inhibit the replication of different closely related viruses within the *Alphavirus* genus, we tried to determine if this compound has broader antiviral activity. To test this hypothesis, we decided to analyze Ginkgolic acid’s ability to affect the replication of the emerging *Flavivirus* ZIKV. To do this, Vero cells were pre-treated with Ginkgolic acid or DMSO as described earlier and infected at different times with ZIKV; then viral progeny production was measured by plaque-forming assay. In DMSO-treated cells, we observed a clear time-dependent increase in viral titers (Figure 6A). In contrast, in the Ginkgolic acid-treated cells, there was an appreciable reduction in viral progeny production in both tested times and this effect was dose-dependent (Figure 6A,B). To verify whether the virucidal activity of Ginkgolic acid observed with CHIKV, MAYV or UNAV also occurred with ZIKV, we incubated 10^5^ PFU of the virus in serum-free medium that contained the drug or DMSO as a control. These experiments revealed again that Ginkgolic acid had a potent inhibitory effect on the virus’ infectivity capacity (Figure 6C). Finally, we performed a time addition experiment as previously described to evaluate in which stage of the ZIKV life cycle the treatment was affecting. As shown in Figure 6D, we found that Ginkgolic acid only affected ZIKV replication when it was administered in the first 2 hpi as we observed in the experiments with the alphaviruses. These findings indicate that Ginkgolic acid also exhibits inhibitory activity against ZIKV and suggests that this compound could have broader antiviral activity.

## 4. Discussion

Emerging arboviruses represent a serious threat to global public health [28]. To date, there are no FDA-approved antivirals to combat these infections and protective vaccines are still being developed. In addition, the spread of several arboviruses to new territories and the increasing availability of arthropod vectors, frequently promotes outbreaks in high-risk regions [29,30]. As a consequence, there is an imperative demand to find new antivirals with potential broader activity.

In this study, we assessed the possible antiviral activity of the natural compound Ginkgolic acid against CHIKV, MAYV, UNAV, and ZIKV. Our results indicate that this drug is capable of reducing replication in a dose-dependent manner in all tested arboviruses. Previous studies suggest that Ginkgolic acid has diverse biological activities, among them, antibacterial [20], antiparasitic [21,31], anti-inflammatory [32] and antitumoral activity [18,19,33]. In addition, other studies have demonstrated that this molecule can inhibit HIV protease in a cell-free system and HIV infection in human peripheral blood mononuclear cells [22,34].

Given that several natural compounds have the ability to directly affect viral particles’ infectivity, we explored the putative virucidal effect of Ginkgolic acid on arboviruses. These experiments show that Ginkgolic acid has a strong effect on infectivity for all the arboviruses evaluated. This is supported by previous reports of other natural molecules, such as Cucurmin, Epigallocatechin gallate, and Luteolin, demonstrating virucidal activity against ZIKV, CHIKV and JEV, respectively [35,36,37].

Natural molecules can disturb viral replication at different stages of the viral life cycle, including viral entry, viral protein expression, assembly and viral release [38,39,40]. With this in mind, we performed a time addition experiment to evaluate the impact of Ginkgolic acid on arbovirus life cycles. Our findings suggest that this compound affects the immediate early or early phase of virus replication. In connection with this, we decided to study alternative mechanisms by which Ginkgolic acid could prevent *Alphavirus* replication. Thus, we assessed the expression of viral proteins E1 and nsP1 using Western blot. These analyses suggest that Ginkgolic acid suppresses the expression of both viral proteins. Other studies have shown that natural compounds, such as Harringtonine can inhibit CHIKV replication through the suppression of viral protein expression.

Although our in vitro results suggest that Ginkgolic acid may have the potential to act as an antiviral drug, its transition toward a therapeutic use presents some important challenges. Some studies have revealed that Ginkgolic acid demonstrates cytotoxic activity at high concentrations in different cell lines [41,42]. Similar findings on cytotoxicity have been reported using animals [43]. However, one pharmacokinetic study showed that Ginkgolic acid can be detected at micromolar levels in rat plasma following oral administration of a small dose (10 mg/kg), suggesting that non-cytotoxic doses of this compound could be used in animal models to evaluate its possible antiviral activity [44]. With respect to this, further detailed studies are required to evaluate the efficacy and safety of this compound as an antiviral drug in an animal infection model. However, to our knowledge, this is the first time that Ginkgolic acid’s broader antiviral activity against different arthropod-borne viruses has been demonstrated.

## Supplementary Materials

The following are available online at https://www.mdpi.com/1999-4915/12/4/449/s1, Figure S1: Cytotoxicity evaluation of Ginkgolic acid in Vero or HeLa cells.
