# Supplementary material for: Broad Antiviral Activity of Ginkgolic Acid against Chikungunya, Mayaro, Una, and Zika Viruses
Source: Viruses. 2020 Apr 15;12(4):449. doi: 10.3390/v12040449 (PMC7232212; doi:10.3390/v12040449)
Supplement: Supplementary file 1 [file viruses-12-00449-s001.pdf]

viruses

MDPI

Supplementary material

Broad Antiviral Activity of Ginkgolic Acid Against Chikungunya, Mayaro, Una and Zika Viruses

Dalkiria Campos [1], Susana Navarro [1], Yessica Y. Llamas-González [1,2] Madelaine Sugasti [1] and José González-Santamaría [1,*]

[1] Grupo de Biología Celular y Molecular de Arbovirus, Instituto Conmemorativo Gorgas de Estudios de la Salud, Panamá 0816-02593, Panamá; dcampos@gorgas.gob.pa (D.C.); nsusana09@gmail.com (S.N.); qfb.y.llamas@gmail.com (Y.Y.L.-G.); madelaine313@gmail.com (M.S.)

[2] Programa de Doctorado en Ciencias Biológicas, Universidad de la República, Montevideo 11200, Uruguay

* Correspondence: jgonzalezsantamaria@gorgas.gob.pa; Tel.: +507-527-4814

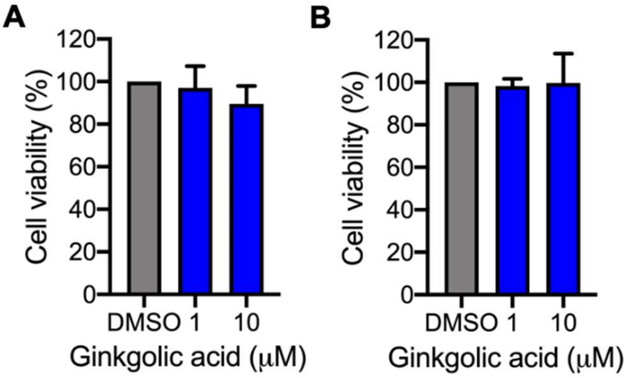

Figure S1. Cytotoxicity evaluation of Ginkgolic acid in Vero or HeLa cells. Vero (**A**) or HeLa (**B**) cells were treated with Ginkgolic acid at indicated concentrations for 24 h, and cell viability was determined using the MTT method. Data were analyzed with the One-way ANOVA test using GraphPad software.
